# The Dual Immunoregulatory Role of *CREB3L1* Underlying Latent and Severe Tuberculosis Clinical Manifestation

**DOI:** 10.1111/imm.70081

**Published:** 2025-12-12

**Authors:** Felipe T. Lima, Ricardo C. Castro, Francisco R. Javier, Caroline Fontanari, Valdes R. Bollela, Rogerio S. Rosada, Célio L. Silva, Lúcia H. Faccioli, Luiz G. Gardinassi, Fabiani G. Frantz

**Affiliations:** ^1^ School of Pharmaceutical Sciences of Ribeirão Preto University of São Paulo Ribeirão Preto Brazil; ^2^ Medical School of Ribeirão Preto University of São Paulo Ribeirão Preto Brazil; ^3^ Ribeirão Preto Nursing School University of São Paulo Ribeirão Preto Brazil

**Keywords:** *CREB3L1*, immune response, monocytes, *Mycobacterium tuberculosis*, susceptibility, transcriptome

## Abstract

During tuberculosis (TB), organ‐specific immune responses and intracellular pathways play critical roles in disease progression and prognosis. Identifying genes that regulate these immune mechanisms remains a key challenge in improving TB management strategies. To investigate genes potentially associated with enhanced resistance to TB and the modulation of immune responses, we analysed RNA‐seq data from whole cells isolated from the lungs and livers of mice infected with *Mycobacterium tuberculosis* (*Mtb*) at two time points that represent different outcomes. We hypothesised that these two organs mount distinct responses to infection, supported by differences in the immune response and bacterial burden kinetics observed in each tissue. Our analysis revealed differential gene expression profiles between the lungs and livers, primarily involving metabolic and immune‐related pathways. Through meta‐analysis, we identified orthologous genes shared between *Mtb*‐infected mice and human patients with latent pulmonary TB. In the omics analysis, the four genes, Creb3l1, Myo7b, Cyyr1, and Cbs, were differentially expressed and associated with either resistance or susceptibility. In vitro assays further demonstrated that knockdown of CREB3L1 in *Mtb*‐infected THP‐1 or primary human monocytes impaired key effector functions, including phagocytosis, bacterial killing, and apoptosis. Taken together, these findings indicate that CREB3L1 possibly contributes to the regulation of genes essential for bacterial control in the lungs during latent TB infection. In contrast, its increased expression in the peripheral blood of patients with severe TB is more likely linked to systemic inflammatory dysregulation rather than direct antimicrobial activity. Notably, CREB3L1 expression in these patients positively correlated with cytokines such as IL‐17, IL‐12, and IFN‐γ, which are central to macrophage activation and effector T cell recruitment. Thus, CREB3L1 appears to play a dual role in TB: under controlled infection, it acts as an immunomodulator limiting excessive pulmonary inflammation, while in severe disease, it may reflect an attempt by the host to amplify inflammatory responses to counteract progressive infection.

## Graphical abstract

1. RNA‐seq of lungs and livers from *Mycobacterium tuberculosis* ‐infected mice with distinct disease outcomes revealed organ‐specific responses driven by differences in immune activity and bacterial burden. Meta‐analysis identified orthologous genes shared with human latent TB and mouse liver, with *Creb3l1*, *Myo7b*, *Cyyr1* and *Cbs* differentially expressed and associated with disease resistance.
2. During in vitro infection of blood‐derived monocytes, CREB3L1 knockdown impaired phagocytosis, bacterial killing, and apoptosis, supporting its role in host defence.
3. Conversely, elevated CREB3L1 expression in PBMCs from TB patients presenting advanced disease correlated with inflammatory cytokines (IL‐17, IL‐12, IFN‐γ). CREB3L1 appears to play a putative context‐dependent role in TB, modulating immunity at the infection site during latency, but amplifying systemic responses in peripheral blood during severe disease.

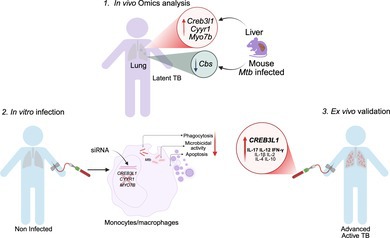

## 1 Introduction

In 2023, the WHO reported that an estimated 1.25 million people died from tuberculosis in the previous year, including 161 000 individuals living with HIV [1]. Although about 90% of those infected have an effective immune response against the bacillus and develop latent tuberculosis, the high numbers related to tuberculosis deaths are entirely due to the active form of the disease. Most incident TB occurs in immunocompetent hosts; however, immunocompromised states (e.g., HIV infection) markedly increase the risk of progression and severe disease [2].

Patients infected by the latent form can potentially become symptomatic and transmitters if the disease is reactivated or remain asymptomatic and undiagnosed for life [3, 4]. This indicates the need to improve the diagnosis and surveillance, and the need to increase our knowledge about tuberculosis [5]. The bioprospection of molecular targets may aid in the diagnosis and/or treatment [6, 7, 8, 9, 10] as well as in understanding the complex immune response against *Mycobacterium tuberculosis* (*Mtb*) infection.

Considering the potential for immune evasion by *Mtb*, successful dissemination through lymphatic or hematogenous routes can lead to infection in various organs beyond the lungs. Among these, the liver is a notable target, along with the spleen and bone marrow [11, 12, 13, 14, 15, 16, 17, 18]. Interestingly, the liver has emerged as a promising site for the identification of prognostic biomarkers, given evidence of its efficiency in controlling *Mtb* infection [19, 20].

Consistent with findings from other groups [20, 21, 22, 23], through the experimental model, we found that bacterial burden is more contained in the liver than in other tissues, indicating that the liver could have specific mechanisms involved in infection control that could be identified. Based on these observations, we hypothesise that comparative analysis of gene expression between the lung and liver may reveal key genes associated with susceptibility and resistance to *Mtb* infection.

Here, after obtaining differentially expressed genes (DEGs), we compared our data with public data from patients diagnosed with L. TB and validated 4 genes: *CREB3L1*, *MYO7B*, *CYYR1*, and *CBS* related to TB control or susceptibility. In vitro analyses demonstrated the impact of these genes on the monocyte response to *Mtb* infection, while ex vivo analyses demonstrated, more specifically, the correlation between *CREB3L1* expression and active tuberculosis.

## 2 Methodology

### 2.1 Animals

BALB/c adult female mice, weighing between 20 and 25 g and about 8 weeks old, coming from the Central Animal House of the University of São Paulo, Campus of Ribeirão Preto—USP, were infected with the H37Rv strain of *Mtb*, according to the Committee on Animal Research and Ethics from the Medical School of Ribeirão Preto (protocol number 020/2012 CETEA, FMRP‐USP). After anaesthesia, the mice were inoculated intratracheally (i.t.) with a suspension of 1 × 10^5^ bacilli/100 μL of *Mtb*. For the determination of the number of colony‐forming units (CFU) kinetics, the animals were euthanized with an anaesthetic overdose at 6, 10, 14, 19, 24, and 31 days after infection. At each time point, 5 animals were euthanized for the kinetics of organ extraction. Then, the organs were processed, and the final solution was diluted and seeded in 7H11 solid culture medium and incubated for 28 days at 37°C. The mycobacterial colonies were counted with the aid of a magnifying glass. The number of colonies was corrected according to organ dilutions and weight and expressed as log^10^ per gram of organ. The lung suspension was diluted 10^2^, 10^3^, 10^4^, and 10^5^ times; the spleen and liver, 10, 10^2^, 10^3^, and 10^4^ times.

### 2.2 Extraction and Evaluation of RNA From Liver and Lung Samples

The right lung of the animals was collected and stored in 1.0 mL of Trizol Reagent (Invitrogen, Carlsbad, CA, USA). The RNA was analysed for its integrity and concentration using the Agilent 2100 bioanalyzer with the RNA LabChip Kit (Agilent Technologies, USA) to certify that it had the characteristics required to be used in RNA sequencing technology (RNAseq).

### 2.3 Processing and Differential Analysis of Gene Expression

The RNAseq was performed using the SOLiD Total RNAseq kit (Applied Biosystems). The material was used for the construction of fragment‐like libraries, for further sequencing using the kits and protocols recommended by the SOLID Platform. Mapping revealed that the coverage achieved by sequencing was 4.5 times the total mouse transcriptome, averaging approximately 7 million reads.

The data obtained through the SOLID platform was initially filtered by a structure designed for the efficient identification of errors within the sequencing of the platform. For this, the quality values (QV) informed by the SOLID primary analysis are used; the parameter used in our filtering was QV ≥ 20, and the objective of this filtering is to obtain data with a high‐reliability index for functional applications.

The raw data were processed using the R language and the environment for statistical computing (R) 3.5 and the Bioconductor 3.9 [24, 25]. The Rsubread, a statistical programme package, was used to align to the reference genome, GRMc38_p6, and to generate a read count table [26, 27, 28].

There were approximately 27 000 mapped genomic features, and reads with very low counts in all libraries were removed from the dataset with the filterByExpr function of the edgeR package. The remaining 18,394 genomic features were normalised to record counts per million (logCPM) with the edgeR package's calcNormFactors function.

### 2.4 Pathway Analysis

To perform the functional analysis, we obtained the Ensembl IDs for the corresponding genome features using the international MGI database (http://www.informatics.jax.org/). Pathway analyses were performed using the ToppGene online database (https://toppgene.cchmc.org/) [29, 30]. We determined significantly enriched pathways by a *p* value < 0.05 and FDR < 0.01. *p* values have been transformed to −Log10 for ease of viewing; therefore, the greater the value of −log10 (*p* value), the greater the importance of enrichment.

### 2.5 Public Data Meta‐Analysis

To perform the meta‐analysis, public microarray data from 11 previously published studies and deposited in the Gene Expression Omnibus public data repository were used (GEO—https://www.ncbi.nlm.nih.gov/geo/). Meta‐analysis was performed from studies using whole blood samples from humans infected with *Mtb*. The study identifiers are: identifiers GSE19435, GSE19439, GSE19491, GSE19444, GSE28623, GSE41055, GSE42825, GSE42826, GSE42830, GSE42834, and GSE83456.

The meta‐analysis was performed using the OMiCC Compendia Commons (OMiCC; https://omicc.niaid.nih.gov) [31]. It is worth mentioning that we considered a minimum number of biological replicates in the studies used in this analysis, in which each group of samples must contain at least three replicates.

The meta‐analysis resulted in a transcriptional signature for latent tuberculosis (L. TB), whose DEGs were filtered by an adjusted *p*‐value < 0.001. To compare the DEGs obtained via OMiCC with our mouse data, we identified orthologous human genes with Ensembl/Biomart (https://www.ensembl.org/). After obtaining the orthologous genes, the lists were crossed using InteractiVenn (http://www.interactivenn.net/), allowing us to identify DEGs that overlapped with the human datasets and the mouse datasets.

### 2.6 Study Groups

Healthy volunteers and patients with active TB were invited to participate in the research, registering their authorization by signing an informed consent form in accordance with approval by the Research Ethics Committee (CEP/FCFRP‐USP: n° 427‐CAAE, n°62362216.8.0000.5403, and n°84557318.1.0000.5403).

The blood samples from patients diagnosed with active TB, with less than 2 months of antibacterial treatment, were collected at the Infectious Diseases Unit of the General Hospital of the Medical School of Ribeirão Preto. For the composition of the two groups, individuals of both genders, aged between 18 and 65 years, and without co‐infection with HIV, hepatitis, or other chronic diseases, were recruited.

Lesions in patients with active TB were classified as minimal (MIN) when they showed mild or moderate density, were located above the second chondrosternal junction, involved only one segment of one or both lungs, and did not exceed the volume of a single lung. In moderate (MOD) disease, lesions were densely confluent but occupied no more than one‐third of a lung, with cavitation not exceeding 4 cm in diameter. Advanced (ADV) disease was defined by lesions exceeding the extent observed in stage 2 [32].

### 2.7 Isolation of PBMC and Monocytes

The human blood samples were centrifuged at room temperature for the collection of the plasma. Then, the cell portion was diluted in Phosphate Buffered Saline (PBS) 1×, and PBMCs were isolated with the density separation technique using Ficoll‐Paque PLUS, *d* = 1.078 g/mL (GE Healthcare Bio‐Sciences AB, Uppsala, Sweden) according to the manufacturer's instructions. For the in vitro infection experiments, PBMCs were quantified, and monocytes (CD14+ cells) were purified using positive selection with magnetic nanoparticles (BD Biosciences) according to the manufacturer's instructions.

### 2.8 THP‐1 Cell Lines

THP‐1 cells were cultured in RPMI 1640 medium supplemented with 10% heat‐inactivated fetal bovine serum, 100 U/mL penicillin–streptomycin, 2 mM L‐glutamine, 2 mM sodium pyruvate, and 10 mM HEPES. To ensure cell protection and reduce possible experimental bias, cells between the fifth and tenth passages were used in the experiments.

### 2.9 Mycobacterium Infection

All procedures involving tuberculous *Mtb* H37Rv (ATCC 27294TM, Rockville, MD) and non‐tuberculous *Mycobacterium bovis* (BCG—Bacillus Calmette‐Guérin) strains were performed in the biological safety room level 3 at FMRP‐USP. The mycobacterial strains were kindly provided to the research by Prof. Dr. Célio Lopes Silva. Third‐generation strains were subcultured in 7H9 liquid medium enriched with TM Middlebrook ADC (BD Biosciences, Sparks, USA) and incubated at 37°C for 11 days until bacterial growth reached the optical density corresponding to 1x10^7^ mycobacteria/mL.

The monocyte suspension was adjusted according to the experiment, as follows: for qPCR experiments: 2 × 10^6^ each well; for siRNA experiments: phagocytosis and killing 7.5 × 10^3^ per well; for apoptosis experiments: 3.5x10^4^ per well. In all experiments, Dulbecco's Modified Eagle Medium (DMEM) Thermo Fisher supplemented with 10% fetal bovine serum (GibcoBRL) was used. After the period previously established for cell adhesion and 4 h, treatment with siRNA, the cells were infected with mycobacteria, with a multiplicity of infection (MOI) of 1:1 for 2 or 24 h.

### 2.10 Microbicidal Activity

Blood monocytes or THP‐1 cells were infected with *Mtb* and incubated for 2 h (phagocytosis) or 24 h (microbicidal activity) in a CO_2_ incubator at 37°C. After this, cells were disrupted through saponin incubation, and 0.5 mg/mL of resazurin was added and incubated for 24 h in the dark. As controls, monocytes were incubated in DMEM and saponin culture medium, and resazurin was added in the absence of bacteria. The reading of resazurin activity was performed in a spectrofluorometer (SpectraMax Gemini XPS, Molecular Devices) with excitation at 560 nm and emission at 590 nm. For the % of microbicidal activity, primary human monocytes or the THP‐1 cell line had their microbicidal efficiency evaluated, with phagocytosis values of 100% and microbicidal activity values as the percentage of bacteria remaining 24 h after infection. Resazurin reduction test values in relative fluorescence units (RFU) were obtained based on the concentration of bacteria per well evaluated.

### 2.11 Expression of Target Genes by Monocytes and PBMC

The extraction of total RNA was performed using the Trizol reagent (ThermoFisher Scientific, Waltham, USA) following the manufacturer's instructions. For real‐time PCR, RNA samples were converted into cDNA using the “High‐Capacity cDNA Reverse Transcription Kit” (Applied Biosystems, Foster City, USA) following the manufacturer's instructions.

The expression of the target genes was analysed by RT‐qPCR for the following genes: *CREB3L1, CYYR1, MYO7B, and CBS*. 50 ng of cDNA and the “2× qPCRBIO SyGreen Mix Hi ROX” mix (PCR BIOSSYSTEMS, Wayne, Pennsylvania, USA) were used, following the manufacturer's instructions for setting the cycles in the thermocycler. The sequences of the primers used are listed in Table 1. The PCR cycling conditions were as follows: 95°C for 10 min, followed by 40 cycles of 95°C for 15 s and 60°C for 1 min. Results were analysed using the 2 − ΔΔCT method. β‐Actin was used as the constitutive control. The reaction was performed using the StepOnePlus system (Applied Biosystems).

**TABLE 1:** Sequences of primers for the target genes.

Gene	Forward (5′‐3′)	Reverse (5′‐3′)
*CREB3L1*	CCACGAGACCACCAAGTACC	GTACCAGGGGTCCGTCCTAT
*CYYR1*	GCTGCTCTCTCCATCTGATCGC	ATTCCAGGCAAGATCGCCCATTG
*MYO7B*	GCTCAGCATCCAGAAAGTCC	CCTGTTGCAGTAGCCTCTCC
*CBS*	CCGACTCAGTGCGGAACTACAT	GTTCCCAAGCGTCACCATTCC
*β‐Actin*	CCAGCCTTCCTTCCTGGGCAT	AGGGCAATGATCTTGATCTTCATT

### 2.12 siRNA Knockdown

The siRNA knockdown was performed with MISSIONsiRNA Transfection Reagent (S1452, Sigma Aldrich) using the MISSION siRNA Universal Negative Control n° 1 (SIC001‐10NMOL, Sigma Aldrich), in addition to the siRNAs for specific target genes, namely: *CREB3L1* (NM_052854, Sigma Aldrich), *CYYR1* (NM_052954, Sigma Aldrich), and *MYO7B* (NM_001080527, Sigma Aldrich) all at 50 nM. The protocol was followed according to the manufacturer's instructions.

### 2.13 Cytokines Detection

Cytokines and chemokines quantification (IFN‐γ, TNF‐α, IL‐17, IL‐4, IL‐2, IL‐1β, IL‐12, IL‐1β, IL‐6, IL‐8/CXCL8, IL‐10, and CXCL10/CRG‐2) was performed on samples of cell culture supernatant from monocytes treated or not with pharmacological compounds and infected or not in vitro with *Mtb*. Additionally, cytokine levels were evaluated in plasma samples from healthy donors and patients with active TB. The detection assays were performed on the multiplex platform using the Magnetic Luminex Assay kit (R&D Systems, a biotech brand, Minneapolis, USA), analysed in a plate reader (Luminex Multiplexing Instrument—EMD Millipore, Luminex Corporation, Austin, TX, USA) according to the manufacturer's instruction manual. All results were calculated by Milliplex Analyst 5.1 software using an individual standard curve for each cytokine/chemokine.

### 2.14 Apoptosis and Necrosis

After the culture, primary human monocytes were resuspended in FVS 780 diluted in PBS and then in PBS with Annexin V (BD Bioscience). After incubation, cells were centrifuged, the supernatant was discarded, and the cells were fixed in formaldehyde and stored. CompBeads Anti‐Mouse Ig, κ/Negative Control Compensation Particles Set (Cat# 552843, RRID: AB_10051478). The selection of the positive population was performed using the FMO (fluorescence minus one) strategy. Data were acquired with BD FACSDiva (100 000 or 200 000 events per sample) and analysis was performed using FlowJo 10.10 software.

### 2.15 Statistical Analysis

Differentially expressed genes (DEGs) were obtained using the moderated t‐test method with the limma package for R [33, 34, 35]. DEGs were selected based on adjusted *p* value < 0.01 by the false discovery method (FDR), and the Fold Change was < −1.5 or > 1.5. Heat maps were generated with log CPM from selected DEGs, while clustering was conducted with Pearson's correlation distance for metric calculations and Ward's binding method, with the gplots package [36].

Principal component analysis (PCA) was performed with R and visualised with the ggbiplot package. Bar graphs for single genes represent mean ± SD and were generated with GraphPad Prism version 9. To compare the means obtained between the different groups in the experiments, statistical analysis was chosen based on the distribution of parametric or non‐parametric data, followed by the D'Agostino‐Pearson omnibus test. The analysis was performed using an unpaired two‐tailed *t*‐test or one‐way ANOVA with Tukey's multiple comparisons test. Pearson correlations were performed in GraphPad Prism version 9. We used the appropriate statistical test for the experiment in question, with significance established at ****p ≤ 0.0001, ***p ≤ 0.0005, **p ≤ 0.005, *p ≤ 0.05.

## 3 Results

### 3.1 The Abundance of Transcripts in Total Cells From *Mtb*‐Infected Organs Demonstrates Different Types of Responses to Infection

**FIGURE 1:**
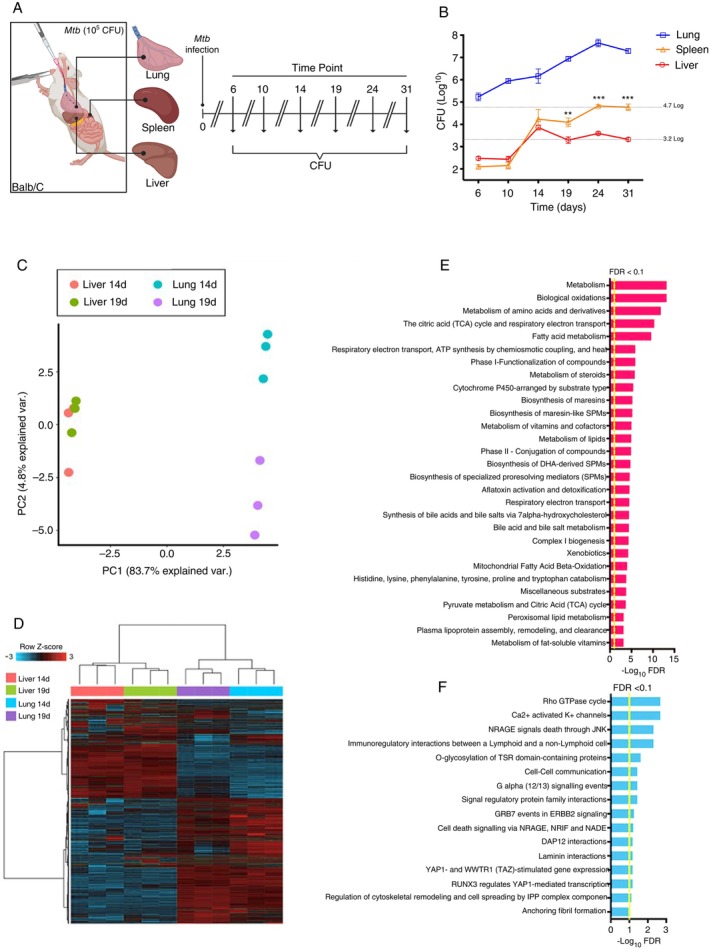
Analysis of transcriptomic data of total liver and lung cells of Mtb‐infected mice. (A) Experimental design of infection kinetics in BALB/c mice with *Mtb*. The mice were infected intratracheally with 10^5^ CFU per animal, and the infection kinetics were compared by assessing CFU from the lung, spleen, and liver at six different time points post‐infection. At each time point, 5 animals were euthanized for the above kinetics organ extraction. (B) Recovery of *Mtb* from lung, spleen, and liver, represented as colony‐forming units (CFU) per gram of tissue, at Days 6, 10, 14, 19, 24, and 31 post‐infection. (C) Principal component analysis (PCA). Expression data from all 18 394 annotated genes were used for this analysis. Genes expressed on Day 14 after *Mtb* infection (pink, liver 14d), genes expressed on Day 19 after *Mtb* infection (green, liver 19d), genes expressed on day 14 after *Mtb* infection (blue, lung 14d), and genes expressed on day 19 after *Mtb* infection (purple, lung 19d ‐). (D) Heatmap of annotated genes for each organ on the 2 days evaluated (14d–19d). (E) Pathways enriched in the ToppGene online database using annotated genes from the initial liver analysis. (F) Pathways enriched in the ToppGene online database using annotated genes from the initial lung analysis. The graph represents the mean ± SEM of the CFU count/g of tissue. Statistical analysis was performed by unpaired One‐way ANOVA. **p < 0.01 and ***p < 0.001.

Initially, infection kinetics was performed to determine when a more efficient liver–lung response to *Mtb* infection would become evident, as indicated in the literature [20, 21, 22, 23]. To define the mediators involved in TB susceptibility and control in mouse tissues, we performed infection kinetics and colony‐forming units (CFU) evaluation between 6 and 31 days. At day 14, described in the literature as an early time point for infection [37, 38], *Mtb* was already established in the lung, and the host immune system was mounting a response. At this point, CFU recovery from the liver and spleen was comparable. However, by day 19, divergent patterns emerged: the liver showed signs of infection control, while the spleen exhibited increasing CFU counts, following the same trend as the primary target organ, the lung (Figure 1A,B). Based on this shift between days 14 and 19, we hypothesised that a specific modulation occurs in the liver that allows bacterial control, in contrast to the lung, where active infection persists. Therefore, we next evaluated gene expression in the liver, as the organ potentially responsible for controlling systemic dissemination, and compared it to the lung, the primary site of infection. To this end, we selected days 14 and 19 post‐infection, the critical time points highlighted by CFU recovery, and performed RNA sequencing to identify transcriptional biomarkers associated with bacterial containment in the liver versus persistence in the lung (Figure 1C).

Seeking to explain how the liver responded more efficiently to *Mtb* infection, we analysed the data generated by the RNAseq technique of total cells of the liver and lung. Because these samples are from different organs, this difference was evidenced through our initial analyses, in which PCA was used (Figure 1C).

In addition, we also used the heatmap to visually observe clusters formed by the liver and lung gene expression patterns. We observe that there is a difference in inter‐organ gene expression at the evaluated time points, indicating the formation of clusters composed of genes that exhibit different expression profiles between organs, demonstrating that there is indeed a distinct response between them to the same process of infection (Figure 1D). Moreover, we characterised the differences of each organ through pathways enriched with genes specifically expressed in each organ. The most significantly enriched pathways in the liver include several metabolic processes, such as biological oxidations, amino acid metabolism, TCA cycle, fatty acid metabolism, and cytochrome P450‐related pathways (Figure 1E). Meanwhile, in the lung, the identified pathways are mainly related to immune signalling, cellular communication, and regulation of cell death. Notable pathways include the Rho GTPase cycle, Ca^2+^‐activated K^+^ channels, NRAGE signalling, and interactions in cell death and immune modulation (Figure 1F).

Furthermore, when we analysed the gene expression profiles of both tissues on days 14 vs. 19 post‐infection, the results showed that infection time significantly impacted gene expression in both organs (Figures S1A and S2A). Unsupervised hierarchical clustering of highly significant DEGs revealed distinct, time‐dependent expression patterns in each tissue (Figures S1B and S2B). In the lung, the pathway enrichment analysis demonstrated that on Day 19, there was upregulation of genes associated with inflammatory responses, reflecting an immune attempt to eliminate the pathogen (Figure S1C). In contrast, pathways related to signalling and gene activation were downregulated (Figure S1D), including genes encoding epigenetic enzymes such as Hdac4, 7, 8 and 9 (Figure S1E). In the liver, upregulated pathways were mainly associated with metabolism, including dermatan sulphate biosynthesis, lysosomal degradation of glycoproteins, insulin regulation, and post‐translational modifications (Figure S2C). Downregulated pathways included those linked to cytochrome P450, which is involved in xenobiotic metabolism, biological oxidation, and fatty acid biosynthesis (Figure S2D). Additionally, it highlights the suppression of specific cytochrome P450 (CYP) family genes on day 19 (Figure S2E).

**FIGURE 2:**
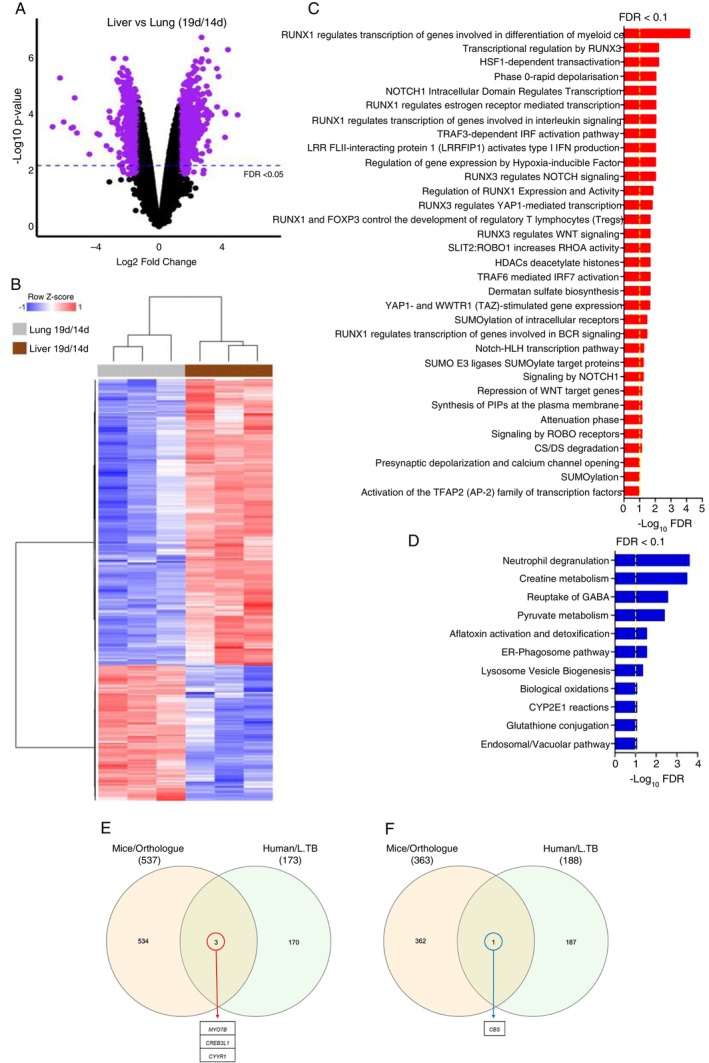
Comparison between transcriptional signature of liver versus lung (19d/14d). (A) Differential expression analysis between states of infection with *Mtb*. Liver vs. lung (19d/14d). The number of DEGs is shown, whereby the purple dots in the volcano plots depict DEGs using an FDR < 0.05. (B) Heatmap of DEGs resulting from liver vs. lung sample analysis comparing 19d/14d after *Mtb* infection. Blue means repression, and red means expression. (C) Pathways enriched in ToppGene's online database using DEGs, upregulated, from liver vs. lung sample analysis. FDR < 0.1 (D) Pathways enriched in ToppGene's online database using DEGs, downregulated, from liver vs. lung sample analysis. FDR < 0.1. (E, F) Venn's diagram showing common genes detected between our analyses, liver vs. lung (19d/14d), and public data derived from patients diagnosed with L. TB. (E) Intersection between upregulated genes in the liver of mice and the lung of L. TB. (F) Intersection between downregulated genes in the liver of mice and the lung of L. TB.

Given the changes related to the progression of the infection, we performed a comparative analysis of the liver versus the lung using gene expression from Day 19 normalised by Day 14 (19d/14d) for each organ. This analysis identified DEGs potentially linked to differential organ responses to *Mtb* (Figure 2A). The heatmap illustrates clear distinctions in gene expression patterns between liver and lung (Figure 2B). Enrichment of up‐ and downregulated DEGs revealed the involvement of key molecular pathways, including NOTCH and TRAF signalling, and the RUNX transcription factor (Figure 2C,D). Notably, upregulated epigenetic mechanisms (Figure 2C) varied between organs, suggesting a role for epigenetic regulation in determining resistance or susceptibility to infection. Together, these results demonstrate the existence of differences in transcript abundance in whole cells from *Mtb*‐infected organs, suggesting the regulation of distinct types of response to infection control, especially evident in the liver.

To deepen our analyses and identify possible genes involved in effector functions in the control of *Mtb* infection, we performed a translational approach. For this, we compared our data with gene expression profiles obtained from whole blood samples of patients diagnosed with latent tuberculosis (L. TB) (Figure 2E,F, and Tables S1 and S2). The intersection between the two groups revealed genes that may be related to tuberculosis infection; in particular, *Creb3l1*, *Cyyr1* and *Myo7b* were upregulated in the liver and also upregulated in whole blood samples from L. TB (2E and Figure S4A). Moreover, we observed genes that were downregulated between groups; the CBS gene makes up the intersection between the liver and L. TB (2F and Figure S4B). As we hypothesise that gene pathways present in the liver could be related to the control of infection in latent infection, we further analysed the role of *CREB3L1*, *CYYR1*, *MYO7B* and *CBS* in *Mtb* control.

### 3.2 *CREB3L1, CYYR1 and MYO7B* Genes Are Involved in Effector Functions in *Mtb* Infection Control

It is known that innate immune cells, including monocytes, play a crucial role in maintaining the systemic inflammatory response and possess functional mechanisms that help regulate the growth of *Mtb* [39]. Therefore, to investigate whether *CREB3L1, CYYR1, MYO7B*, and *CBS* genes play a role in modulating functional mechanisms of monocytes in combating infection by virulent *Mtb* strains, we evaluated the phagocytic capacity and microbicidal activity of infected monocytes (Figure 2A, Figure S3A).

**FIGURE 3:**
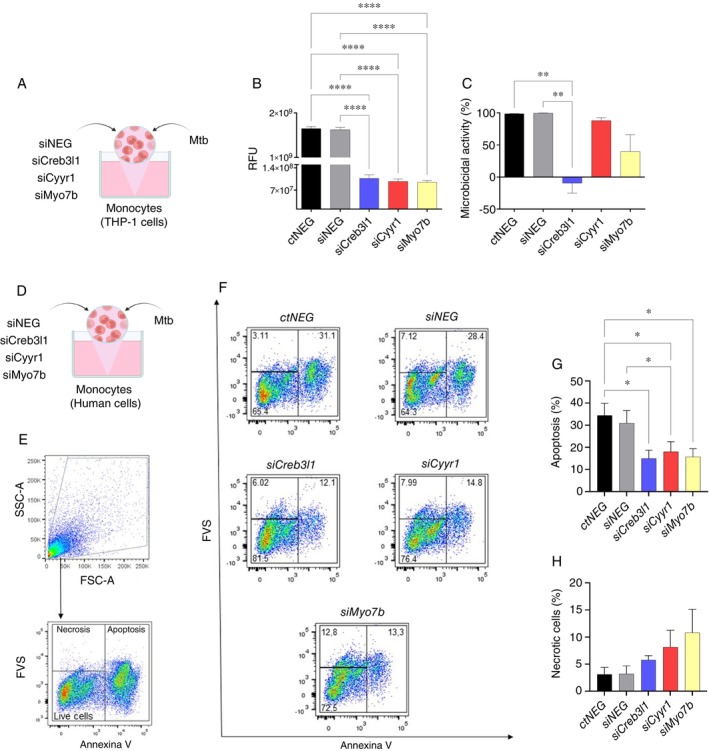
*CREB3L1, CYYR1, and MYO7B* genes modulate monocyte effector functions during *MTB* infection. (A) Experimental design: THP‐1 cells were plated at a concentration of 7.5 × 10^3^, knocked down for 96 h with *siCREB3L1*, *siCYYR1*, and *siMYO7B* with concentrations of 50 nM. After knockdown, monocytes were infected with the virulent *Mtb* H37Rv strain using the MOI ratio of 1:1. Phagocytic activity was assessed after 2 h of culture, while microbicidal activity was analysed after 24 h of infection by resazurin metabolism assay. (B) Phagocytic and (C) microbicidal activity of THP‐1 cells after *Mtb* infection. (D) Experimental design: Primary human monocytes from healthy donors were plated at a concentration of 7.5 × 10^3^, knocked down for 96 h with *siCREB3L1*, *siCYYR1*, and *siMYO7B* with concentrations of 50 nM. After knockdown, monocytes were infected with the *Mtb* H37Rv strain using the MOI ratio of 1:1. After 24 h, the apoptosis and necrosis processes were evaluated through annexin V labelling and subsequent detection by flow cytometry. (E) Flow cytometry gating strategy for cells labelled/stained with Anexin V/Fixable Viability Stain (FVS). (F) Representative flow cytometry plots showing the percentage of cells undergoing apoptosis and necrosis. (G) Percentage of cells undergoing apoptosis. (H) Percentage of cells undergoing necrosis. (A–C) *N* = 4 and (D–H) *N* = 3. The bars represent the mean ± SEM of each group. Statistical analysis was performed by unpaired One‐way ANOVA to perform a multiple comparisons test. *p < 0.05, **p < 0.01, and ****p < 0.0001.

Initially, we validated the downregulation of *CREB3L1, CYYR1*, and *MYO7B* in primary human monocytes and THP‐1 monocytic cells (Figure S3A–E). Suppression of *CREB3L1, CYYR1*, and *MYO7B* genes by siRNA knockdown resulted in a reduction in the phagocytic capacity of THP‐1 monocytes (Figure 3B). Notably, the absence of *CREB3L1* markedly impaired microbicidal activity (Figure 3C). In primary human monocytes, although a similar pattern of impairment in phagocytic and microbicidal capacities was observed, these changes were not statistically significant (Figure S3F–H). The same was observed in cultures of primary human monocytes treated with the pharmacological compound Triamcinolone for induction of the *CBS* gene, in which no significant differences in these response mechanisms were detected either (Figure S3I–K). The biological heterogeneity of samples, donor‐to‐donor variability, and differences in basal gene expression levels can attenuate detectable knockdown effects in primary cells [40]. We recognise this as a limitation of our work, while emphasising that the data presented here are consistent with the current literature on the technical challenges of siRNA delivery in primary cells [41]. On the other hand, although we did not observe differences in phagocytosis processes and microbicidal activity, cells treated with triamcinolone showed a modulation in the production of pro‐ and anti‐inflammatory cytokines and chemokines (Figure S4). We observed a decrease in the levels of TNF‐α, IL‐8, and IL‐2 (Figure S4B,C,E), while there was an increase in the levels of CXCL10 and IL‐10 (Figure S4D,F).

In TB, apoptosis cell death mechanisms are associated with a better resolution of the infection compared to necrosis [42], so we evaluated the patterns of cell death (Figure 3D–H). We observed that suppression of *CREB3L1*, *CYYR1* and *MYO7B* genes specifically resulted in reduced apoptosis in primary human monocytes, but did not alter the necrosis (Figure 3G,H). Taken together, these results suggest that the genes evaluated, especially *CREB3L1*, play a key role in the immune response of monocytes against *Mtb*, modulating phagocytosis, microbicidal activity, and apoptosis.

### 3.3 *CREB3L1* Expression Is Associated With Higher Plasma Cytokine Levels in Patients With Active TB

**FIGURE 4:**
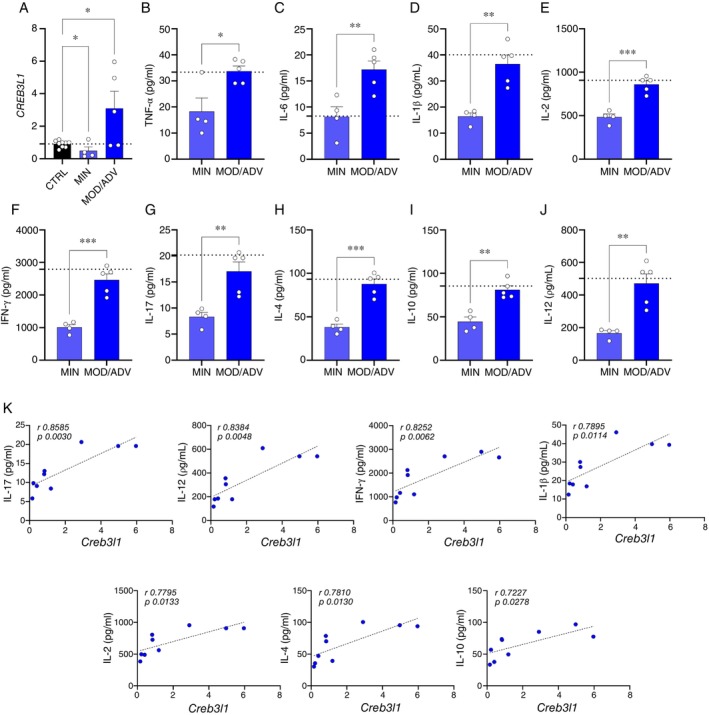
*CREB3L1* expression in the PBMC from patients with active TB is associated with higher plasma cytokine levels. (A) Gene expression of *CREB3L1* in PBMC from healthy donors (CTRL) and patients with active TB with radiological severity score minimal (MIN) and moderate/advanced (MOD/ADV). (B–J) Cytokine levels were evaluated in plasma samples from patients with active TB MIN and MOD/ADV through the Magnetic Luminex Assay Kit. CTRL group (*N* = 9) and patients with active TB MIN (*N* = 4) and MOD/ADV (*N* = 5). (K) The expression of *CREB3L1* on PBMC cells from patients with active TB (MIN + MOD/ADV) was correlated with plasma levels of IL‐17, IL‐12, IFN‐γ, IL‐1β, IL‐2, IL‐4, and IL‐10. The bars represent each group's mean ± standard deviation, and each spot represents an individual. Statistical analysis was performed by unpaired One‐way ANOVA, performing multiple comparisons test or two‐tailed *t*‐test. ***p ≤ 0.0005, **p ≤ 0.005, *p ≤ 0.05.

Given that the absence of *CREB3L1* in monocytes leads to the failure of phagocytosis and killing *Mtb*, as well as is related to unbalanced cell death, but its expression in the lung of L. TB and in the liver of mice seems to be related to TB resistance in vivo, we hypothesised that *CREB3L1* is an immune regulator gene. Then, we investigated whether this factor is expressed in circulating blood cells from patients with active tuberculosis. Interestingly, we found that *CREB3L1* expression is significantly increased in PBMCs from patients with more severe forms of the disease, while it was reduced in patients with less severe forms (Figure 4A), reinforcing our idea that *CREB3L1* can be an immune regulator gene. Furthermore, when analysing the cytokine levels in these patients, we observed higher concentrations of TNF‐α, IL‐6, IL‐1β, IL‐2, IFN‐γ, IL‐17, IL‐4, IL‐10, and IL‐12 in those with higher *CREB3L1* expression (Figure 4B–J).

Finally, our correlation analysis revealed that increased levels of *CREB3L1* in PBMCs from patients with active tuberculosis are positively correlated with higher levels of IL‐17, IL‐12, IFN‐γ, IL‐1β, IL‐2, IL‐4, and IL‐10 (Figure 4K). Among these, IL‐17, IL‐12, and IFN‐γ stand out, as they are essential cytokines for the control of *Mtb* infection due to their central role in the activation of protective immune responses, such as macrophage activation and recruitment of effector T cells (Figure 4K).

These findings suggest that *CREB3L1* can be a key immune regulatory factor related to the balance between protective immunity and immunopathology during tuberculosis. Its role appears to be context‐dependent and site‐dependent, while its absence impairs fundamental antimicrobial functions at the site of infection, its increased expression in non‐infected cells in patients with more severe disease coincides with heightened inflammatory responses. This is a dual role of *CREB3L1* acting as a fine‐tuning of the immune response to achieve effective bacterial control.

## 4 Discussion

Genome‐wide transcriptional profiling has been an instrumental technology in deciphering the host response to *Mtb* infection [43, 44]. It is also possible to observe how cells from different tissues present a different transcriptomic profile when facing the same infection by *Mtb* [45]. Thus, in this work, we established a model to study susceptibility and immunopathology by comparing the genes involved in the response to TB. For this, we made a parallel between the transcriptome of the liver and lung during experimental infection by *Mtb* and in the latent and active TB infections.

Following intratracheal inoculation, the *Mtb* bacilli reach the alveolar space in the lungs and subsequently enter the systemic circulation through lymphatic drainage or disruption of the alveolar‐capillary barrier. Once in the bloodstream, the bacilli disseminate hematogenously and can get to other organs, such as the liver and spleen. In our experimental model, the early presence of bacteria in extrapulmonary tissues likely reflects hematogenous dissemination, rather than true colonisation. This interpretation is supported by previous work showing that *Mtb* disseminates via the bloodstream early after infection, and organ‐specific immune responses determine whether bacteria are contained or proliferate [46, 47]. To minimise bias in interpretation, we report CFU starting from Day 6 and focus comparisons between Days 14 and 19, when infection is fully established.

Until Day 14, bacterial loads in the liver and spleen are comparable, reflecting similar exposure via the bloodstream. However, by Day 19, the spleen bacterial burden increases approximately 1 log higher than in the liver, highlighting the superior ability of the liver to control bacterial replication. Both organs were exposed to circulating bacteria, but only the liver effectively limited infection. In contrast, by Day 31, the spleen and lung show progression towards active infection, and we can observe an increase of 1.5 log of CFU in the spleen compared to the liver. Thus, we choose to work with our transcriptome data comparing the liver with the lung precisely to be able to visualise the difference between these organs that respond so differently to the same infection. Eliminating as much as possible the natural difference between the organs, we sought to observe only the effects caused by the infection.

The liver and lung have their physiological functions well known, and the consequent importance for the organism is evident. However, because they perform different metabolic and physiological activities, they are expected to respond differently to an infectious process, and we were able to observe this difference at the transcriptional level.

By identifying pathways and mechanisms activated in the hepatic response that more efficiently controlled *Mtb* infection, we believe that this organ can serve as a basis for the identification of new biomarkers related to protection or susceptibility to tuberculosis [22, 48, 49, 50], as well as KORF et al., 2009, who, using an animal model, identified the “Liver X receptors” (LXRs) as modulators of inflammation and that are closely related to lung cells in what they believe to be a previously unknown function for these nuclear receptors in a mechanism of resistance to *Mtb* infection [22]. Our comparative transcriptomic analysis between liver Day 19 and lung Day 14 post‐*Mtb* infection revealed clear divergence in gene expression patterns, as evidenced by heatmap clustering. Functional enrichment analyses implicated key signalling pathways, such as NOTCH, TRAF, and RUNX, in mediating these differences. Interestingly, our group has already demonstrated the influence of the Notch pathway in the human tuberculosis progression and immune response activation, in which NOTCH1 and DLL4 were involved in the TB control [32].

We then compared our analyses with data from samples from L. TB. The idea of this comparison is to observe if there are genes commonly expressed in samples from the lungs of patients considered resistant to infection and thus identify possible molecules or pathways related to resistance in the liver. The intersection showed us that there are 3 upregulated genes (*Creb3l1, Cyyr1*, *Myo7b)* and 1 downregulated gene (*Cbs*). These genes have been reported in the literature as playing a role in the *Mtb* infection process [51, 52, 53, 54]. We also observed that the effector functions of *Mtb*‐infected THP‐1 or primary human monocytes are especially impaired in the knockdown of the *CREB3L1, CYYR1*, and *MYO7B* genes.

The *Cyyr1* (Cysteine and Tyrosine‐Rich Protein 1) is not extensively studied, while the *Myo7b* encodes an unconventional myosin implicated in cell–cell communication and solute transport [52]. Given that the execution phase of apoptosis involves actin–myosin‐dependent processes such as membrane remodelling, cell contraction, and nuclear fragmentation, the downregulation of *Myo7b* may interfere with apoptotic execution by disrupting cytoskeletal dynamics [55]. Therefore, CYYR1 (poorly characterised) and MYO7B (actin‐dependent motor) may modulate apoptotic sensitivity without directly affecting bacterial clearance within the assessed time frame. We now frame this as a biological divergence in downstream functional consequences and highlight it as a limitation that merits further investigation in the field.

Among the validated genes, *CREB3L1* seems to be of particular interest because of its strong capacity to interfere with bacterial killing. Its absence reduced the phagocytosis capacity, abrogated the control of infection, and unbalanced apoptosis/necrosis induction. There are reports in the literature that support our observation [56], showing the role of the transcription factor *Creb3l1* in the modulation of genes involved in the stress response of the endoplasmic reticulum in the rat hypothalamus. Endoplasmic reticulum stress response is directly related to apoptosis [57], This shows us, from another perspective, the importance of this gene in the process of *Mtb* infection, since apoptosis is a favourable mechanism to control *Mtb* infection because it induces the activation of innate and adaptive immune responses, and cell necrosis is directly linked to the spread of *Mtb* [58, 59].

In a complementary way, the triamcinolone treatment to induce the *CBS* [60] gene expression modulated a set of pro‐ and anti‐inflammatory genes after the *Mtb* infection. In support of our findings, Saini et al. demonstrated the importance of the *Cbs* gene during *Mycobacterium tuberculosis* infection. Using mice deficient in cystathionine β‐synthase (Cbs), an enzyme responsible for producing hydrogen sulphide (H₂S), the authors observed that these animals exhibited prolonged survival and reduced mycobacterial burden in infected organs. Moreover, the same study showed that pharmacological inhibition of *Cbs* expression similarly led to a reduction in *Mtb* bacterial load in mice [61].

Interestingly, the expression levels of TNF‐α, a key cytokine involved in apoptosis induction [62], appeared to be lower compared to the controls; at the same time, anti‐inflammatory cytokines, like IL‐10 and CXCL10, were more expressed than in the same controls. Therefore, specific modulation of this gene would possibly have an impact on the immune response against *Mtb*, especially considering that its expression was reduced in L. TB, as observed in omics analyses.

Our experimental findings suggest a putative functional role for *CREB3L1* in host defence. Notably, its increased expression in the lungs of L. TB patients and the livers of *Mtb*‐infected mice further supports its involvement in resistance mechanisms in vivo. These converging lines of evidence led us to propose *CREB3L1* as a potential immune regulatory factor in tuberculosis. To explore this hypothesis further, we examined its expression in circulating blood cells from patients with active TB, segregated by severity, and observed that the more severe the condition of patients, the more *CREB3L1* is expressed. At first, this seemed contradictory, since higher expression correlated with worse disease outcomes. However, tuberculosis immunopathology involves a fine‐tuning regulation of cytokines and cell activation depending on the infection status of the cell.

*CREB3L1* is an endoplasmic reticulum (ER) stress sensor and a transcription factor strongly associated with apoptosis control, but it also plays a direct role in other pathologies. *CREB3L1* is activated in response to infection by viruses (such as HCV, West Nile, Sendai, and herpesviruses), undergoing cleavage and releasing its N‐terminal domain, which translocates to the nucleus and induces the expression of cell cycle inhibitory genes. This limits the proliferation of infected cells, helping to contain viral spread [63]. In patients with pancreatic cancer, *CREB3L1* promotes M2 polarisation, which reduces the TCD8+ infiltration, favouring the tumour progression [64]. Another study, exploring 33 different cancers in the Cancer Genome Atlas (TCGA) database, evidenced its abnormally high or low expression depending on the cancer type and its role as an immunoregulator when associating with the presence of infiltrating leukocytes [65].

Given that *CREB3L1* regulates the expression of genes involved in the endoplasmic reticulum stress response, which can influence immune function, particularly under conditions of chronic inflammation, infection, or cancer, here we propose that, in the lung during *Mtb* latent infection, *CREB3L1* contributes to the regulation of genes essential for controlling bacterial growth. In contrast, its high expression in peripheral blood cells from severely infected TB patients reflects systemic inflammatory dysregulation rather than direct antimicrobial activity, as the blood is not the primary site of infection. This interpretation is further supported by the cytokine levels we observed in these patients, where CREB3L1 expression positively correlated with IL‐17, IL‐12, and IFN‐γ, cytokines known for their central roles in macrophage activation and effector T cell recruitment [66]. Thus, in severe disease, peripheral CREB3L1 may indicate an attempt by the host to amplify inflammatory responses to control bacterial replication in the lungs, whereas in controlled infection (L. TB), CREB3L1 appears to adopt an immunomodulatory role, limiting excessive pulmonary inflammation. A limitation of our study is the absence of direct comparative analyses between peripheral blood and pulmonary compartments, including cells isolated directly from bronchoalveolar lavage fluid, as well as comparative transcriptome profiling of blood cells and liver from infected mice. Addressing these aspects in future work will be essential to fully elucidate the context‐dependent roles of CREB3L1.

## Author Contributions

F.T.L. and R.C.C. recruited the participants, performed the conceptualization, conducted experiments, data curation, formal analysis, wrote the original, reviewed, and edited the manuscript. R.S.R. performed the conceptualization and conducted animal model experiments. C.L.S. and L.H.F. provided reagents and methods, and funding acquisition, and edited the manuscript. L.G.G. supervised the bioinformatic data analysis and reviewed the final manuscript. F.G.F. performed the conceptualization, funding acquisition, resources, project administration, supervision, and data curation, reviewed, and edited the manuscript.

## Funding

This study was funded by the Coordination for the Improvement of Higher Education Personnel (CAPES), Financial code 001, and by São Paulo Research Foundation (FAPESP) (grant numbers 2011/07455‐8, 2018/15066‐0, and 2018/14968‐0).

## Conflicts of Interest

The authors declare no conflicts of interest.

## Supporting information


**Data S1:** Supporting Information.

## Data Availability

The data that support the findings of this study are openly available in Gene Expression Omnibus at https://www.ncbi.nlm.nih.gov/geo/, reference number GSE299836.
