# Supplementary material for: The Dual Immunoregulatory Role of CREB3L1 Underlying Latent and Severe Tuberculosis Clinical Manifestation
Source: Immunology. 2025 Dec 12;177(4):810–23. doi: 10.1111/imm.70081 (PMC12952985; doi:10.1111/imm.70081)
Supplement: Supplementary file 1 — Data S1: Supporting Information. [file IMM-177-810-s001.docx]

**Supplementary material**

***
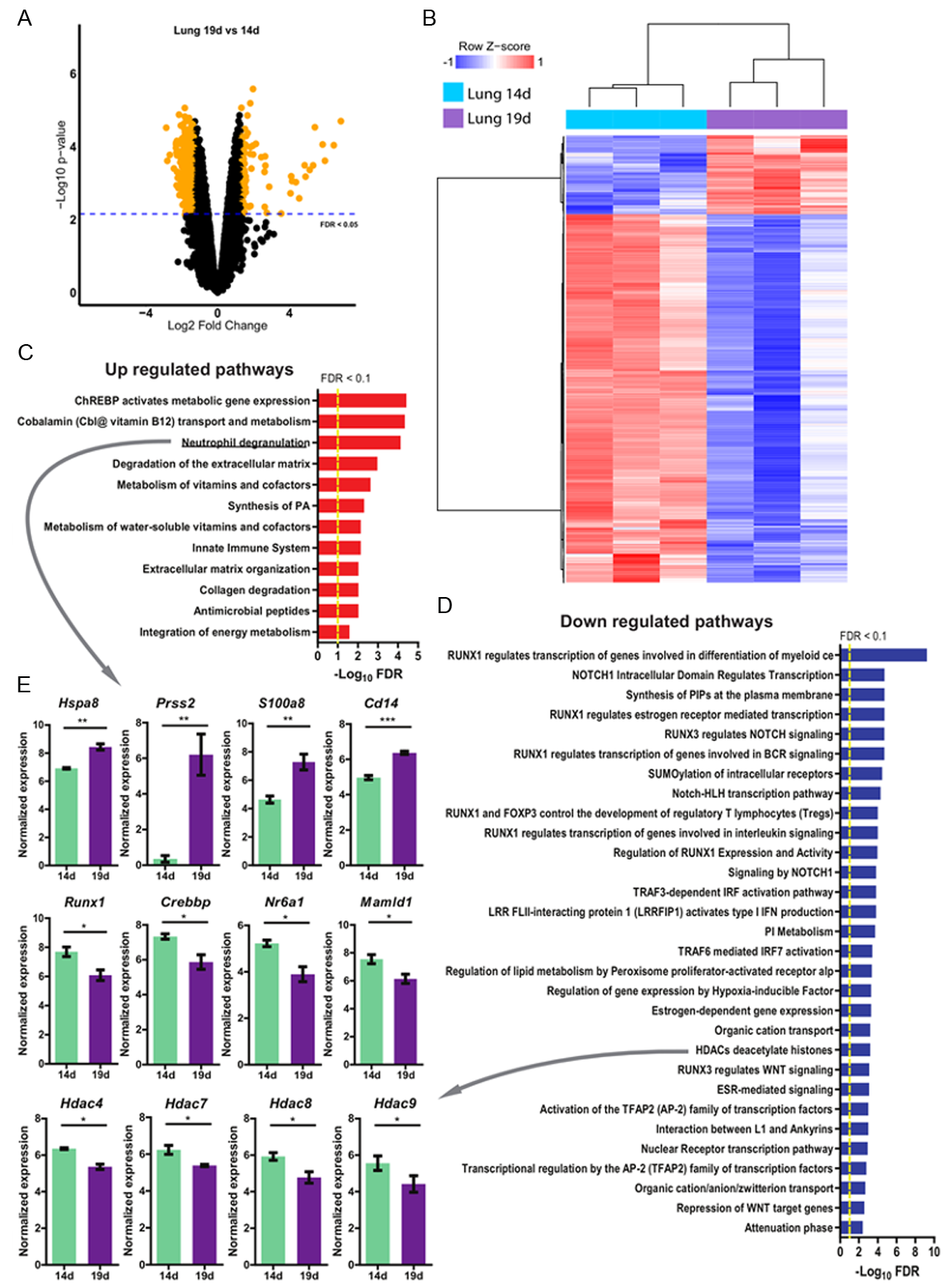
***

***Figure S1. Transcriptional signature of the lung during Mtb infection.*** (A) Differential expression analysis between 19 days vs 14 days of infection with *Mtb*. The number of DEGs are shown, whereby the orange dots in volcano plots depict DEGs using an FDR <0.05. (B) Heatmap of DEGs resulting from lung sample analysis comparing 19d-14d after *Mtb* infection. Blue means repression, and red means expression. (C) Metabolic pathways enriched in ToppGene's online database using DEGs, upregulated, from lung sample analysis. FDR <0.1 (D) Pathways enriched in ToppGene's online database using DEGs, downregulated, from lung sample analysis. FDR <0.1. (E) Individual genes that compose the enriched pathways (compared on days 19d and 14d after infection with *Mtb*.). Based on the normalized raw data count. Statistical analysis was performed by an unpaired two-tailed t-test to evaluate differences among independent genes. ***p ≤ 0.0005, **p ≤ 0.005, *p ≤ 0.05.


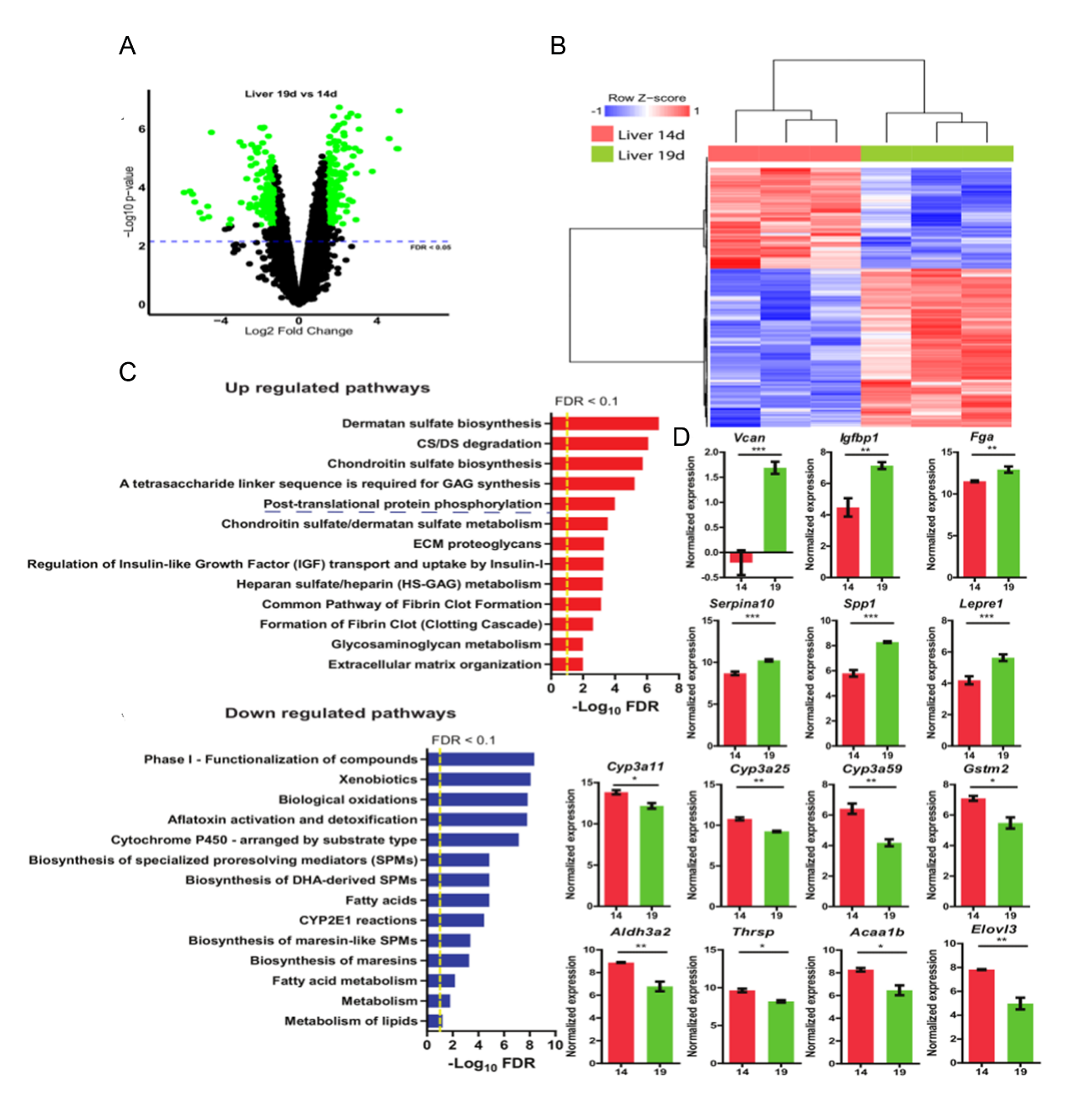


***Figure S2. Transcriptional signature of the liver during Mtb infection*** (A) Differential expression analysis between tissues from *Mtb* infected mice (19d-14d after infection). The number of DEGs are shown, whereby the green dots in volcano plots depict DEGs using an FDR <0.05. (B) Heatmap of DEGs resulting from liver sample analysis comparing 19d-14d after *Mtb* infection. Blue means repression and red expression. (C) Pathways are enriched in ToppGene's online database using DEGs that are upregulated from liver sample analysis. FDR <0.1 (D) Pathways enriched in ToppGene's online database using DEGs, downregulated, from liver sample analysis. FDR <0.1. (E) Individual genes that compose the enriched pathways (compared on days 19d and 14d after infection with *Mtb*). Based on the normalized raw data count. Statistical analysis was performed by an unpaired two-tailed t-test to evaluate differences among independent genes. ***p ≤ 0.0005, **p ≤ 0.005, *p ≤ 0.05.


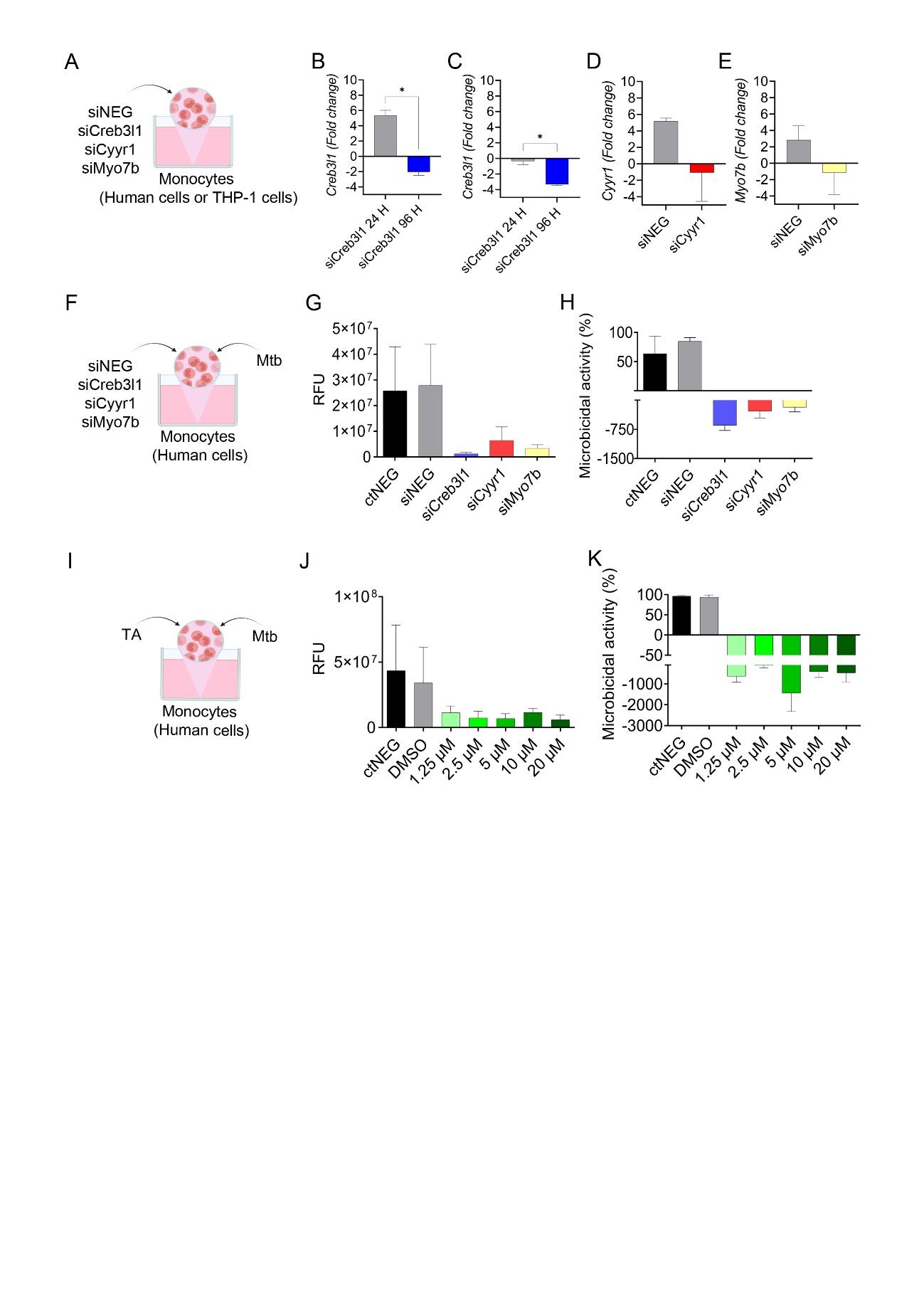


**Figure S3. Evaluation of the phagocytosis and microbicidal activity of primary human monocytes infected with H37Rv after knockdown using siRNA for the *CREB3L1, CYYR1, and MYO7B* genes or treatment using the pharmacological compound Triamcinolone for induction of the *CBS* gene**. (A) Experimental design: primary human monocytes from healthy donors or THP-1 cells were plated at a concentration of 7.5 × 10^3^, knocked down for 24 or 96 hours with *siCREB3L1*, *siCYYR1*, and *siMYO7B* with concentrations of 50nM. Gene expression of *CREB3L1* in the (B) primary human monocyte cells and (C) THP-1 cell lysate 24 or 96 hours after transfection. Gene expression of (D) *CYYR1* and (E) *MYO7B* in the primary human monocyte cell lysate 96 hours after transfection. (F) Experimental design: primary human monocytes from healthy donors were plated at a concentration of 7.5x10^3^, knocked down for 96 hours with *siCREB3L1*, *siCYYR1*, and *siMYO7B* with concentrations of 50nM. After knockdown, monocytes were infected with the *Mtb* H37Rv strain using the MOI ratio of 1:1. Phagocytic activity was assessed after 2 hours of culture, while microbicidal activity was analyzed after 24 hours of infection by rezasurin metabolism assay. (G) Phagocytic and (H) microbicidal activity of primary human monocytes after *Mtb* infection. (I) Experimental design: primary human monocytes from healthy donors were plated at a concentration of 7.5x10^3^ and treated for 24 hours with Triamcinolone at different concentrations. After treatment, the monocytes were infected with the *Mtb* H37Rv strain using the MOI ratio of 1:1. Phagocytic activity was assessed after 2 hours of culture, while microbicidal activity was analyzed after 24 hours of infection by resazurin metabolism assay. (J) Phagocytic and (K) microbicidal activity of primary human monocytes after *Mtb* infection. (A-E) N=2, (F-H) N=3, (I-k) N=3. The bars represent the mean ± S.E.M. of each group. Statistical analysis was performed by an unpaired two-tailed t-test or a One-way ANOVA to perform multiple comparisons test. *p ≤ 0.05.


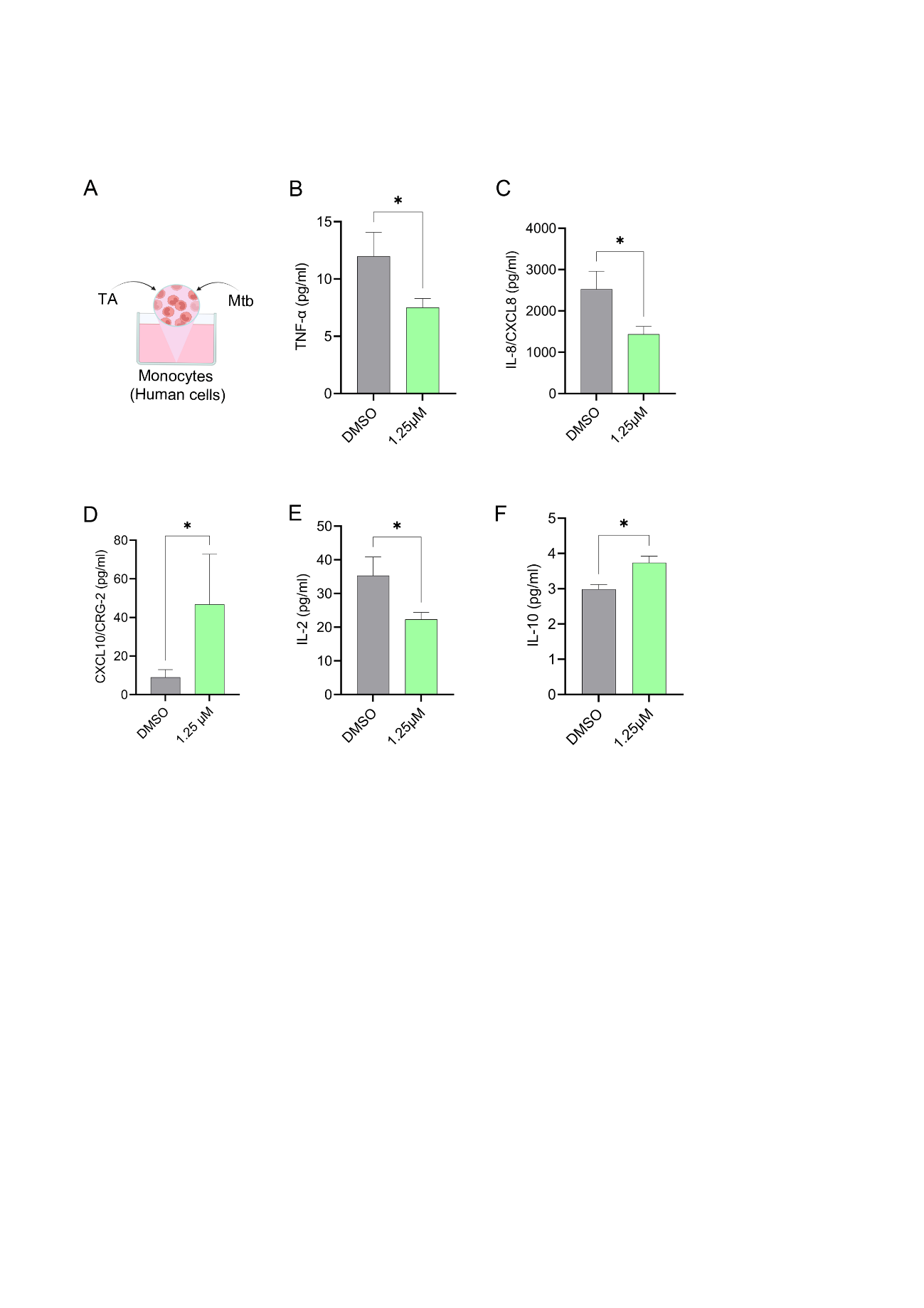


**Figure S4. Quantification of cytokine levels in triamcinolone-treated monocyte culture supernatant against *Mtb* infection.** (A) Experimental design: primary human monocytes from healthy donors were plated at a concentration of 3,5x10^4^ and treated with triamcinolone (1.25µM) for 24 hours. After that, monocytes were infected with the *Mtb* H37Rv strain using the MOI ratio of 1:1. After 24 hours, supernatant in culture medium was collected for evaluating cytokine levels. (B-F) Cytokine levels were evaluated in samples of monocyte culture supernatant in culture medium. Cytokine levels were evaluated through the Magnetic Luminex Assay Kit. (A-F) N= 5. The bars represent the mean ± standard deviation of each group. The results were analyzed using an unpaired two-tailed t-test. * P < 0.05.

Table S1. The relation of upregulated genes in the liver of mice and in the lung of L. TB as shown in the Venn diagram in Figure 2E.

| Signature UP/HUMAN | Signature UP/ MOUSE/ Orthologue |
| --- | --- |
| AADAC, ABCC3, ADAM29, ADD2, AGXT, AIM1L, AMBP, AMIGO1, ARMCX1, ART1, BCAR3, BCL11B, BPNT1, BSND, BTBD16, C12orf28, C17orf74, C17orf97, C1orf187, C1QC, C1QTNF3, C1S, C21orf56, C22orf26, C5orf54, C9orf85, CADM1, CAGE1, CCDC132, CCDC27, CD70, CDC7, CEP55, CHN1, CHODL, CLCN3, CPNE6, CREB3L1, CUBN, CYYR1, DLC1, DPY19L4, DUSP4, EARS2, EGR2, EID3, EMP2, FAM81B, FBLN2, FCN2, FLJ26850, FPR3, FSTL3, GALNT1, GALNT8, GDAP1L1, GHRHR, GLDC, GPR119, GPR15, GSG2, GTSF1L, HAPLN2, HELLS, HGF, HLA-DPB1, HOXB7, HRK, HS3ST1, HSD17B3, HTR1D, IFNG, IGFALS, IKZF3, IL12A, KCNJ4, KIAA1704, KIF21A, KIF24, KRT20, LDHC, LHX1, LRCH2, LRP2, LRRC23, LRSAM1, MAS1L, MCAT, MCC, MELK, MGAT3, MMAA, MMP14, MMP16, MORN3, MOXD1, MSC, MTUS1, MYLK2, MYO1B, MYO7B, MYSM1, NCAPG2, NLGN1, NLRP7, NPTX1, NR5A1, NUAK1, OR1C1, OR1N2, OR2H2, OR51D1, PABPC5, PADI2, PALLD, PCDH9, PCDHB14, PCSK1N, PDE4C, PHTF2, PIK3C2A, PTGR1, PTPN13, QRSL1, RBP5, RDM1, ROR1, RUNDC3B, SCD5, SCEL, SCN1B, SGCE, SHH, SHISA4, SLAMF7, SLC22A16, SLC44A3, SLC7A3, SLC9A6, SPA17, SPACA3, SPATS2L, SPEG, SPRY2, SRR, SYT1, TAS2R8, TFEC, TLL2, TMED6, TMEM128, TMEM19, TMTC1, TNFAIP6, TNFRSF13B, TNIP3, TRIM17, TRNP1, TSPAN15, TSSK1B, TTK, TUBGCP3, TYMS, UBE2U, UGT8, UNC5C, WEE1, WISP2, YY1AP1, ZBTB6, ZNF214, ZNF287, ZNF365 | Lmcd1, Fam206a, BC049987, mt-Tr, Itgb3bp, 0610009E02Rik, Gm10030, Serpina7, Gm12353, Gm43682, Triqk, Klhl29, 4933406C10Rik, Ap1ar, Opn3, Pde6h, Gm31793, Igfbp1, Tox, Gm38048, Gm14398, Bbs1, Setd1a, 2610035D17Rik, Mir17hg, Cerkl, Ighv7-3, Rnft2, Fgl1, Hunk, Alpk1, Ccdc66, Gm48530, Car12, Gm9917, Gstcd, Tm4sf20, Gm30694, Wdr25, Gm20513, Gm28039, Zfp788, Als2cr12, Tmem117, Gm32856, Myo7b, Aspm, Slc41a2, Itfg2, Ltbp3, Map6, Scn3a, Fam222a, Ntrk3, Vegfc, Mybl1, Capn8, Rad51, Cntrl, Gm8883, Parp16, Irs2, Slc41a3, Nhsl1, 2810408I11Rik, Greb1l, Gm13307, Epha7, Gm43573, 9430037G07Rik, Cdk5r1, Gm35339, Gm20507, 9330159M07Rik, Ano6, Tmem189, Tmem39a, Caprin2, Ppargc1a, AC102496,1, B3gntl1, Stag1, Ccdc173, Bbs9, Gm48199, Gm46516, Gm19619, Gm15448, Pfkfb1, 5830408C22Rik, Garem1, Cherp, Spats1, Gm6225, Acpp, Gm16973, Gm11008, Smim14, Chrm3, Il7, Fhad1, Gm14966, Lef1, Mdm1, Dctn6, Ttll8, Prelid2, Cyyr1, Cpne8, Zeb1, Pcgf2, Ppfia2, Vcan, Cdc42ep5, Gm32171, Gm17276, Cep170, Itln1, Xrcc4, Oip5, Ppp4r4, Zfp583, Ccdc92, Synj1, Cfap100, Lmbr1, Aug-03, Slc17a9, Gm14409, Fndc1, Heatr9, Creb3l2, Cep97, Sycp2, Tmem263, Pprc1, Ticrr, A230072C01Rik, Serpinb8, Stox2, 2010310C07Rik, 4930432K21Rik, Spice1, Gm37352, Gm48693, Ppl, Camk4, Rpgrip1l, 6030498E09Rik, Fgg, Sncaip, Fam185a, Lpgat1, Pwwp2a, C030034L19Rik, Fam169b, A530020G20Rik, Sntb1, Dph6, Ralgps2, Sema6a, Dnah17, Jpx, Mamld1, Neb, Pigf, Fhl3, Slc39a12, Adcy3, Ap1s3, Kmt2d, D630045J12Rik, Erc2, Mad1l1, Prtn3, Ubap1l, Col12a1, Zfp366, Riiad1, Gm9317, 3110039I08Rik, Sema6d, Unc45b, B3galt1, Kcnmb2, Gm16867, Prkg1, Lca5l, Copg1, Raph1, Mcf2l, Gm36660, Kif23, CT010445,1, Mbnl2, Pdss1, Mllt3, Trp53inp1, Fhit, Themis, AC161258,2, Nfxl1, Dlg1, Nme7, Slc13a5, Rad51b, EU599041, Col1a1, AC131339,2, 2810403D21Rik, Hbs1l, Orm2, Tet1, C2cd5, Kcnh1, Gm38393, Gm527, Ap3s1, Mid1, Nedd4l, Mir142hg, Pld1, Apoa4, Pgbd1, Meis2, Zgrf1, Ldb2, Gm26973, Cd247, Kcnab1, Gm29811, Garnl3, Gm34220, Gm28198, A230057D06Rik, Exoc6b, Cstf3, 4930417O13Rik, Phf14, Gm6088, Wnt5b, Noct, Zdhhc14, Farp1, Gm6418, Efna5, Rngtt, Pde3a, Hyls1, Gm13710, Runx1, Ccne2, Gm14442, Ttc28, Fam78b, Rai1, Pdlim1, Ppm1h, Fndc3b, Prr12, Fbxl7, mt-Ty, Rps6ka3, Kifc1, Pspc1, Hdac4, Esr1, Speer6-ps1, Gm47715, Abtb2, Adcy1, Ntf3, Nrap, Fyb, Slc9a9, Pla2g4c, Dach1, Agap1, Scfd2, Ppp1r12b, Nnmt, Agap2, Sybu, A630052C17Rik, Vrk2, Gm27003, L3mbtl3, Stxbp6, Rbm25, Fgb, Glmn, 43527, Bcl2, Tesk2, Iqsec2, 4930590J08Rik, Nlk, Nup98, Mertk, Stap1, Siah1b, Arl15, Phex, Sec31a, Pde7a, Tsg101, Gm17231, Acnat2, Gm4258, Junos, Fto, Cmya5, Gm18103, St7, Apbb2, Ccnjl, Scai, Faim, Cdyl2, Gm18815, Map3k5, Hivep3, Pianp, Tmem131, Mybpc1, 4933432I03Rik, Pvt1, Fam117a, Tbc1d8, Pdlim3, Gm29085, Cdyl, Camkmt, Aven, Gm11427, Arhgef25, Rapgef2, Sik3, Gm17203, Cep250, Plcl1, Ust, Zeb2, Grk5, Itgb1bp2, Ano5, Pinx1, Fgd6, Zc3hav1l, 3632454L22Rik, Ppp2r2a, Phip, Slc24a4, Ms4a13, Rfx7, Havcr2, Elp4, Myrfl, Ly6d, Ngf, Lncpint, Sec16a, AW554918, Arhgap32, Chsy3, Btg3, Tcf12, Zfp783, Ylpm1, Smyd3, Fam222b, Gm16008, Apcs, Prkce, Platr22, Osmr, Exoc4, Eno4, Prg4, Slc8a1, 1700113A16Rik, Ankrd44, Cntnap1, Sebox, Fam84a, Gm26621, 5830417I10Rik, Rsrc1, Cntln, 5430402O13Rik, Prickle2, Tcof1, Bora, 4930443O20Rik, 2610307P16Rik, Mllt10, Gm43379, Zswim6, Gtf2e2, Cd46, Gm12610, Rreb1, Pgpep1l, Maml3, 9530026P05Rik, 2310014F06Rik, Pip4k2a, D030028A08Rik, C9orf72, Gm45799, Med12l, Plcb1, Saa4, Zfp14, Zfp831, Gm19325, Gm15027, Acmsd, Cdkal1, Kcnip2, Gale, Rnf32, Zbtb20, Gm20732, Spata17, Hcfc1, Kmt2a, 2610507I01Rik, Gm26871, Kcnt2, Gm12781, Zfpm2, Serp2, Zfp607b, Gm41077, Mkl2, Ep400, Plcxd2, Whrn, Proser1, Maml2, Mitf, Enox2, Thada, Adam22, Ccdc122, Rbm26, Etnk1, Babam2, Khdrbs1, Ccnd3, Srgap2, Efcab10, Zcchc7, Ints6, Dnajb5, Tpte, Zc3h13, Ikzf2, Gm28375, Gm12655, Hectd2, Fut8, Gm28694, Gli3, BC052040, Ppp2r5e, Gm5871, Tcerg1, Nbea, Gm18204, Sgip1, Zfp281, Atp1a3, 4933406I18Rik, Tmem212, Arl5c, Irak1bp1, Gm9733, Xrra1, Cpeb3, Ube2e2, Muc6, Alg13, Supt3, Hdx, Stxbp5, Helz, Prrc2c, Foxp1, Nr4a3, Gm31166, Znrf3, Sox5, Celf2, Gm12194, Fyn, Ica1l, Gpr39, Mkl1, Gm8013, Rnf38, Dync2li1, Actr3b, Ube2l3, 2700099C18Rik, Sec24a, Ahi1, Fbxl17, Mfsd2a, Magi3, Elmo1, 9530052E02Rik, Ank2, Ankef1, Magi1, Ssbp2, R3hdm1, Slx4ip, Creb3l1, Atxn7l1, Trerf1, Baz2b, 1700030F04Rik, Gtdc1, Clnk, 4930579C12Rik, Wasl, Scara5, Rbm6, Rpl31-ps4, Ptprm, Nucb2, Prkca, Gm6695, Wdr17, Cd3e |

Table S2. The relation of downregulated genes in the liver of mice and in the lung of L. TB as shown in the Venn diagram in Figure 2F.

| Signature/Down/Human | Signature/Down/Mouse/Orthologues |
| --- | --- |
| ACOT12, ACSS2, ADAMTS14, AIF1L, ANKRD33, ANKRD53, ANXA7, ARG1, ARPC2, BCL6, BPI, BTNL8, C2orf40, C9orf41, CA3, CA4, CACNG3, CAMP, CASS4, CBS, CCL25, CD300E, CDCA2, CDH16, CECR2, CES3, CLTB, CNKSR1, CNOT1, CRISP1, CRISP3, CYP26B1, CYP27A1, CYP3A43, CYP4F12, DAOA, DARC, DLX2, DMRT2, DNAJB4, DOC2B, DRD5, DSC1, DYNLT3, DYRK3, EFCAB3, ENKUR, ERAP2, ETAA1, EXOC6, FAM118A, FAM174A, FAP, FGD2, FGF11, FHIT, GABRP, GAD1, GALNT14, GCAT, GDPD1, GEM, GHSR, GIMAP6, GOLGA7, GPR37, HAPLN1, HIP1, HKDC1, HOXB2, HP, IL1R2, IL34, IL4, IL8, IMPDH1, INTS6, IQCB1, ITLN1, KANK2, KCNJ10, KCNJ3, KCNK7, KCNN2, KIAA1324, KRT23, KRT72, LCN2, LPO, LRRC6, MAGEB2, MCTP1, MEST, MGAM, MME, MPHOSPH10, MRGPRF, MRPL3, MRPL44, MYBPH, NEUROG1, NKX2-3, NKX3-1, NPHS1, NXF3, OLR1, OPLAH, OPTC, OR10H3, OR11A1, OR1S2, OR2B2, OR3A2, OR8D2, ORM1, ORM2, OSBPL7, OXER1, PADI1, PADI4, PAQR9, PCDHB11, PCDHB9, PCP4, PHC2, PLSCR4, PNPLA1, POSTN, PPIG, PPM1L, PRG2, PRKCD, PTGDS, PTGS2, PVRL2, PXDNL, RARRES2, RCN3, RETN, RHOXF1, RP1L1, RPAP3, RPS27L, RUNX3, SCN3B, SELT, SERPINC1, SH3GL3, SHD, SKIL, SLC13A3, SLC29A1, SLC5A12, SLITRK1, SLITRK5, SNX16, SOAT2, SOX7, SPARCL1, SPTBN1, SSB, STYK1, TACR1, TAS2R5, TCN2, TECPR2, TEK, TENC1, TFCP2L1, TFDP3, TFF3, TGM3, THAP5, TLR3, TMEM176A, TNFAIP8, TNRC6B, TPST1, TRAT1, TRERF1, UBN1, VAX2, VWCE, WDR76, WNT2B, YIPF4, ZNF483, ZNF544 | 2900041M22Rik, Ighv5-12, Gm7774, Ctrb1, Tmem254c, Gm37301, Cel, Ltb, Ccl21c, Pnlip, Ptgir, Ighv1-12, Try5, Gp2, Cela3b, Prss2, Rnf186, Hes1, Ccl20, Try4, Gm5409, 4933406B17Rik, Guca1a, Clic6, 5730416F02Rik, Tm4sf5, Cpa1, Ntf5, Hist1h2ae, Gm5652, Tnfaip2, Cbs, Amy2a5, Pim1, 2210010C04Rik, Gm35106, C1qb, Clps, C1qa, Gm8767, Ube2d4, Odf3b, Amy2a2, Klf10, Tnf, Gm13777, Cpb1, Try10, Ifi30, Fut7, Fam210b, D630039A03Rik, Id1, Mmp2, Prss1, Amy2a4, Gm7289, Suox, Plekho1, Gm14652, Cox6b2, Igkv4-50, Tlcd1, Cela3a, 6820408C15Rik, Gm7436, 1110065P20Rik, Tm4sf1, Hist1h2bp, mt-Tm, Pnliprp2, Ctrc, Sfn, Ctrl, Pnliprp1, Calca, Gm11730, Col7a1, Gm26980, Npc2, Hal, Arrdc3, C1qc, Amy2a1, Gm5678, AC159187,1, Slc38a11, Ccdc33, Pdk4, Slamf9, 1700016K19Rik, Pheta1, Traf1, H2-M10,6, Ctss, Rab19, Acsm3, Cdhr4, Adam15, Thrsp, Ftl1, Clec11a, Igkv4-61, Aldoart2, Gm2343, Gm28071, H2-Aa, Fpr1, Dusp1, Tacc3, Gm2199, Sftpd, Gm19680, Gm5771, Sftpa1, Gm9826, Tyrobp, Creg1, Spock2, Ccdc175, Slc22a18, Cd209g, Krt7, Rpl31-ps18, Rnase1, Sftpc, Slc11a1, Csad, H2-Ab1, Mapk15, Amy2a3, Lox, Vnn1, Cd3g, Slc6a12, Rtl8a, Ywhaq-ps2, Clec4e, Calhm6, Reg1, Ccdc92b, Hk3, Rtl8b, Slc39a2, Insyn1, Sit1, Gm6012, Ap1m2, Ano8, Gm12015, Foxj1, Ldhb, Ctsd, Gm5224, Ckb, Hsd17b6, Gm6579, Gm14032, 2610528J11Rik, Nupr1, Hist1h2bk, H2-Eb2, Lyz2, Psap, Gm7543, AC139573,1, Dusp28, Gpihbp1, Gm2965, Tmem100, H2-DMb2, Cd74, Gm14719, Hist1h1e, Gm13305, Apoe, Gm48733, Spon2, Ly6c2, Lyz1, Prss3, Ptms, Eva1b, Gm8724, Cxcr3, Gm4798, Chil3, Ace, Serpinb1a, Gm2412, Gm9129, Coasy, Ppp1r2-ps4, Wfdc1, Fbln1, Gm4935, Vldlr, Sec14l2, Elovl3, Dnase2a, Cyp4a32, Bmf, Glns-ps1, Gm5389, H2-DMb1, Gm18551, Gm4737, Ighv5-1, Gsta4, Gm8532, Ces2b, Lgals3, Gm18957, Gm49213, Fblim1, Nkx2-1, Epn3, Tff2, Acads, Wfdc17, Npnt, Prdx6b, Nradd, Ctsw, Gm12009, Gm7823, Coq8a, Akr1c14, Gstm2-ps1, Acot2, Tymp, 9130409I23Rik, Acaa1b, Gm4835, Atp5g1, Cela1, Trpm3, Gm16437, Gm8238, Glo1, Irgm2, Cldn5, Inmt, Rps2-ps5, Nkg7, Acot1, Acta2, Ighm, Nrn1, Decr2, Gm13443, Retsat, Gm12856, Abi3, Gstt2, Cyp2e1, Slc25a10, Gm5787, Acsl1, Cyp4b1, Tmem54, Psmb5-ps, Ubxn10, Car1, Tinagl1, Rdh16, Gm2996, Gm29041, Tsc22d1, Igfbp2, Sp3os, Ap3s1-ps2, Gm20452, Gm13503, Tead2, Gm7232, Atp2a3, Chp2, Gm16391, Cyp3a41b, Cyp2a21-ps, AC102410,1, Hmgb1-rs17, Car3, P2rx1, Adora1, Gm12494, Id3, Pdia2, Klb, Ms4a7, Eln, Gm48685, Cyp3a11, Slc26a4, Mphosph8, Zfas1, Mettl7a1, Gjc1, Ager, Gm7856, Ehhadh, Cacnb3, Socs1, Lpcat4, Gm14130, Osgin1, Ccdc184, Gnb5, Mgp, Cyp2c38, Cdkn1c, Gm19109, Gstm4, H2-DMa, Gm17794, Fam83b, Gm16001, Hist1h4a, Spaca9, Cyp2a5, H2-Eb1, Aif1, Smim24, Gstm2, Aldh1b1, Abcd2, Ndrg2, Hba-ps3, Gm7634, Arhgef39, Serpini2, Cyp2a4, Hist2h4, Aldh3a2, mt-Ti, Pdcd1, Cpa2, Gm18276, Aox3, Cyp3a41a, AC123951,1, Gm36758, Gm3222, AC115797,3, Fibin, Cib2, Cfap206, Ggt1, Gm7541, Gm8770, Atf3, Lppos, Acvrl1, Bcam, Serpina6, Tnfrsf19, Ces1f, Fam167b, Lamc2, Cyp4a31, Dcstamp, Gm28729 |
